# Network-based metabolic characterization of renal cell carcinoma

**DOI:** 10.1038/s41598-020-62853-8

**Published:** 2020-04-06

**Authors:** Nishtha Pandey, Vinay Lanke, P. K. Vinod

**Affiliations:** 1 Center for Computational Natural Sciences and Bioinformatics, International Institute of Information Technology, Hyderabad, 500032 India; 2 TCS Innovation Labs, Hyderabad, India

**Keywords:** Biochemical networks, Systems analysis, Network topology, Gene regulatory networks

## Abstract

An emerging hallmark of cancer is metabolic reprogramming, which presents opportunities for cancer diagnosis and treatment based on metabolism. We performed a comprehensive metabolic network analysis of major renal cell carcinoma (RCC) subtypes including clear cell, papillary and chromophobe by integrating transcriptomic data with the human genome-scale metabolic model to understand the coordination of metabolic pathways in cancer cells. We identified metabolic alterations of each subtype with respect to tumor-adjacent normal samples and compared them to understand the differences between subtypes. We found that genes of amino acid metabolism and redox homeostasis are significantly altered in RCC subtypes. Chromophobe showed metabolic divergence compared to other subtypes with upregulation of genes involved in glutamine anaplerosis and aspartate biosynthesis. A difference in transcriptional regulation involving HIF1A is observed between subtypes. We identified E2F1 and FOXM1 as other major transcriptional activators of metabolic genes in RCC. Further, the co-expression pattern of metabolic genes in each patient showed the variations in metabolism within RCC subtypes. We also found that co-expression modules of each subtype have tumor stage-specific behavior, which may have clinical implications.

## Introduction

Major biological processes namely reproduction, development, wound healing and tissue regeneration require cell proliferation. Cells proliferate in response to growth-promoting stimulus however, under adverse conditions they move into a reversible, non-proliferating state termed quiescence. Cells gauge the strength of proliferative and anti-proliferative signals through multiple molecular players to make cellular decisions. Cancer is a proliferative disease that arises when the regulatory control of quiescence-proliferation reversible transition is lost. An emerging hallmark of cancer is metabolic reprogramming, which helps to meet the energy demand for cell growth and division. Initial studies by Otto Warburg pointed to aerobic glycolysis, however recent advances have started to reveal other metabolic alterations and plasticity of cancer metabolism^1,2^. Understanding the differences in metabolism between normal and cancer cells can shed light on the adaptations that promote disease progression and may also facilitate the identification of therapeutic metabolic targets.

Mutations or epigenetic alterations in cancer can influence the expression of metabolic genes. Studies have explored transcriptome data of different cancers to understand the transcriptional dysregulation of metabolic genes. These studies are based on data generated by The Cancer Genome Atlas (TCGA) program. A pan-cancer analysis of different cancer types found a convergent metabolic landscape with upregulated nucleotide synthesis and downregulated mitochondrial metabolism as the main features^3^. Rosario *et al*.^4^ analyzed the gene expression of metabolic pathways in Kyoto Encyclopedia of Genes and Genomes (KEGG) and found that pentose and glucuronate interconversions (PGI) is significantly dysregulated in many cancer types while the polyamine synthesis is uniquely upregulated in prostate adenocarcinoma (PRAD). Peng *et al*.^5^ identified metabolic subtypes in 33 cancer types based on seven major metabolic processes. These metabolic subtypes showed clinical relevance and association with somatic drivers.

A recent study on TCGA data revealed that the classification of 33 cancer types is dominated by tissue-type or cell-of-origin differences^6^. This provides a basis for a focused pan-cancer analysis of individual tissues to map the cancer subtype-specific changes in the metabolism. Renal cell carcinoma (RCC) is a heterogeneous cancer with major histological subtypes including clear cell (KIRC), papillary (KIRP) and chromophobe (KICH). These RCC subtypes differ in the cell-of-origin with clear cell and papillary originating from cells of proximal convoluted tubule while chromophobe originating from cells of distal convoluted tubule of the nephron^7^. Recently, we showed that the site-of-origin dominates the classification of these subtypes using deep learning and histopathological images^8^. A multi-platform genomic data analysis on RCC also showed the site of origin as one of the major determinants in the classification of these subtypes^7,9^. Molecular characterization of RCC revealed the subtype-specific mutations, methylation, and pathways^10^. RCC subtypes have distinct glycolytic and mitochondrial gene expression patterns. A metabolically divergent (MDD) group with poor survival is identified in KICH^10^. The histologic review also reclassified some KIRC samples as KICH^10^. Other studies on RCC specifically focused on metabolic alterations of KIRC^11,12^. The metabolic network of KIRC is associated with chromosome 3p loss of heterozygosity^12^. A comprehensive metabolic characterization of RCC subtypes specifically less common KICH and KIRP is required since most of the pan-RCC studies^9,10^ focus on analyzing the expression patterns within the tumor and/or restrict to selective metabolic pathways.

Genome-scale metabolic models (GEMs) provide a comprehensive view of metabolism and serve as a scaffold for interpreting high throughput data^13^. Network-based approaches have revealed the systems-level alterations of specific cancers and led to the reconstruction of cancer GEMs^14–16^. To further obtain insights into the metabolism of RCC subtypes, a network-based analysis was performed by integrating transcriptomic data with the human genome-scale metabolic model. Our study revealed the role of amino acid metabolism and redox homeostasis in RCC subtypes in-addition to glycolysis and TCA cycle alterations. A difference in glutamine metabolism is observed between subtypes, which is linked to the difference in transcriptional regulation involving HIF1A. The analysis showed that E2F1 and FOXM1 are other major transcriptional activators of metabolic genes in RCC. Further, we also identified metabolic modules that are linked to clinical traits of RCC subtypes based on the co-expression pattern of genes.

## Results

### RCC shows high variation in metabolism compared to other cancer types

**Figure 1:**
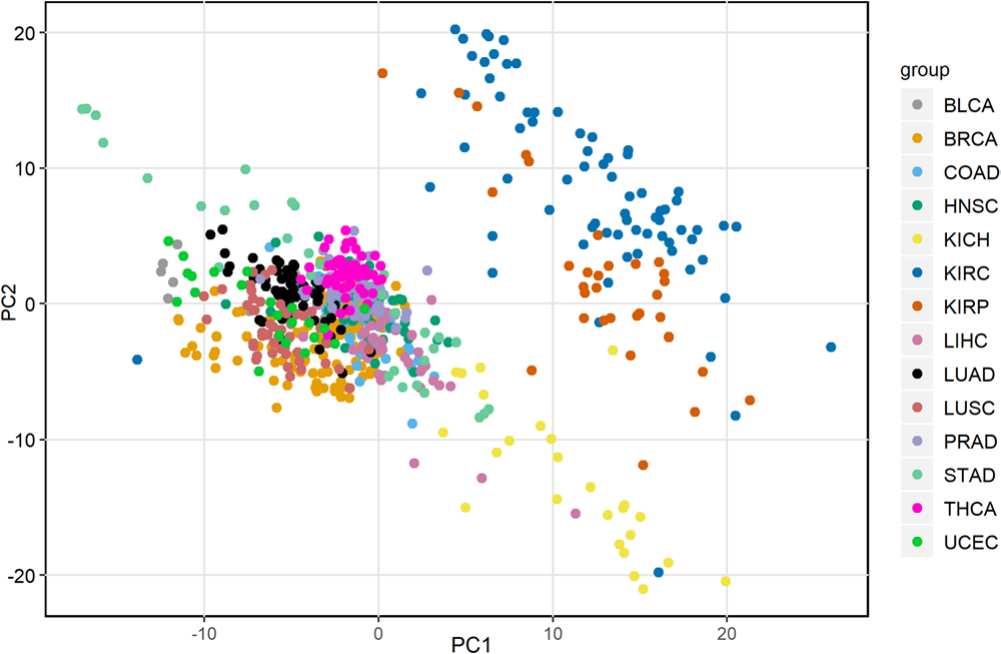
Principal component analysis (PCA) of 14 cancer types. The log fold-change in expression of highly varying genes (361) between matched normal and tumor samples was used to perform the PCA.

We screened 14 cancer types from TCGA (Table S1) based on the availability of RNA-Seq data of both tumor and tumor-adjacent normal samples (668 samples). The human genome-scale metabolic model HMR2 (see methods) was used to study the metabolic differences between matched normal and tumor samples. The relationship between different cancer types was explored based on the fold-change in the expression of HMR2 genes between matched normal and tumor samples. This was done to eliminate the tissue-of-origin differences between cancer types. The principal component analysis (PCA) revealed that RCC samples have high variance compared to other tumor samples (Fig. 1). RCC samples separated into two sub-groups corresponding to RCC subtypes. Further, KIRC and KIRP samples clustered together compared to KICH.

### Reporter metabolic pathways in RCC subtypes

**Figure 2:**
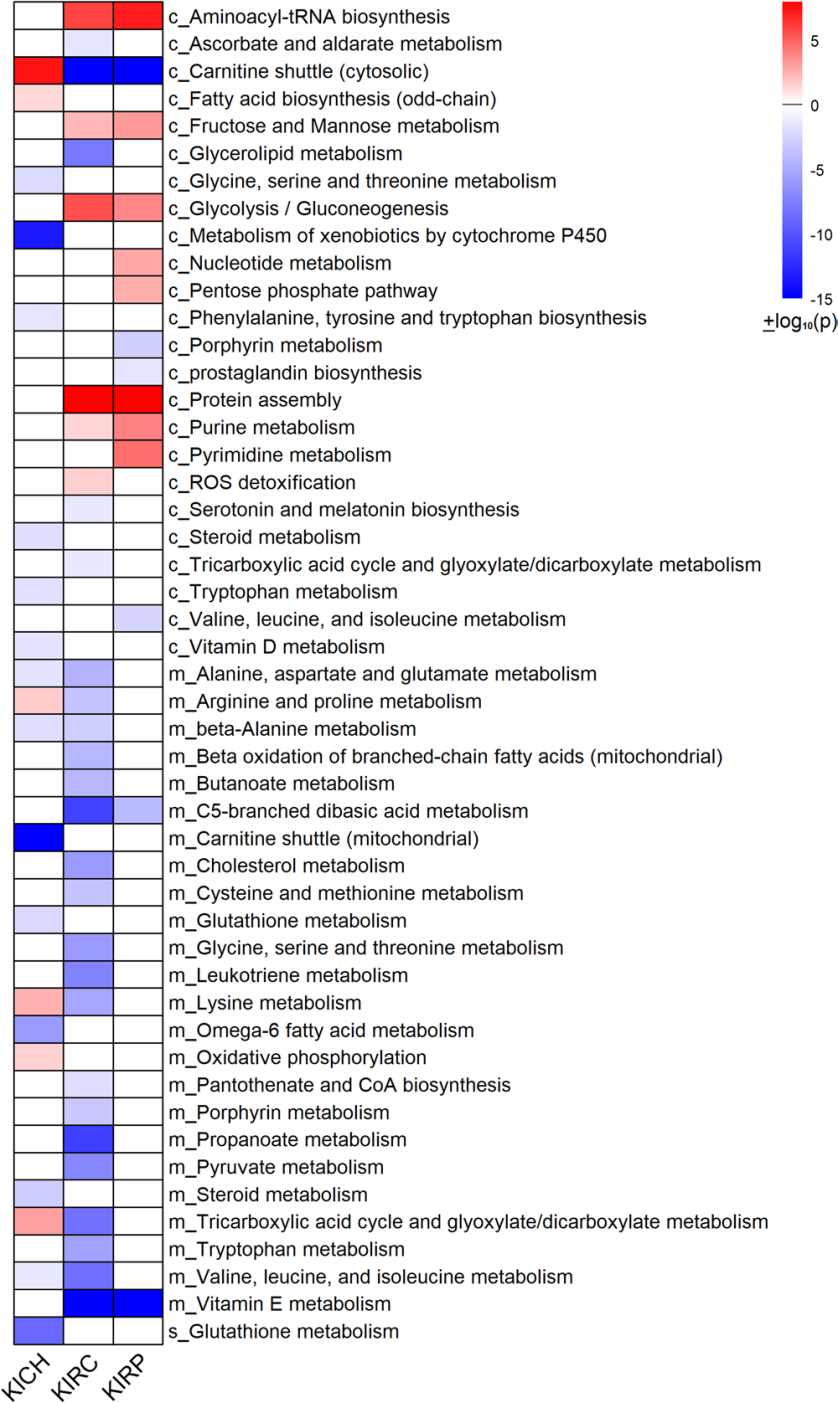
Reporter pathways of RCC subtypes. The upregulated pathways are shown in red and downregulated pathways are shown in blue. The cellular compartment is specified as prefix c, m and s corresponding to cytosol, mitochondria and extra-cellular, respectively. p-values are log transformed (−log_10_*p*) and minus (−) represents the downregulation of pathway.

The differential gene expression (DGE) analysis between matched normal and tumor samples showed that metabolic genes were predominately downregulated in RCC subtypes (Table S2). We performed the transcriptional factor enrichment analysis of differentially expressed genes^17^. The downregulated genes were associated with HNF4A, LXR, RXR and PPARA in RCC (adj p-value <0.05, Table S3). The upregulated genes were associated with E2F1 and FOXM1 in RCC and with HIF1A in KIRC and KIRP (adj p-value <0.05, Table S4). The FOXM1 expression level was higher in late stage samples of KIRP and KICH while the E2F 1 expression level was higher in RCC (data not shown). We characterized the metabolic network-based alterations of RCC by mapping the gene expression changes to the reactions in HMR2 and identified reporter metabolites and pathways^18,19^. Figure 2 shows the reporter pathways of KICH, KIRC, and KIRP (Data S1).

### One carbon metabolism

**Figure 3:**
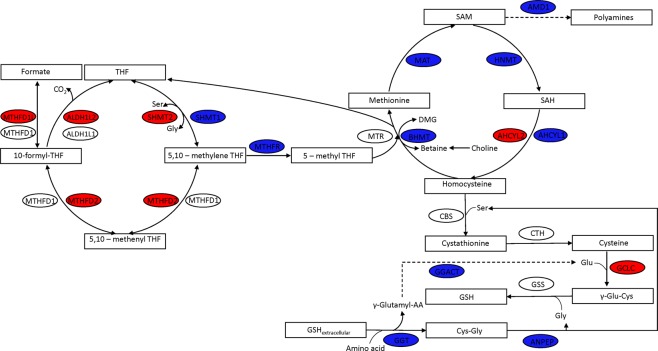
One carbon metabolism is affected in KICH. The expression of genes involved in folate cycle, methionine cycle and glutathione synthesis is altered. Downregulated genes are shown in blue while upregulated genes are shown in red colour.

We found that the glycine, serine and threonine metabolism was downregulated in KICH. Serine and glycine provide one-carbon units to the folate cycle through one-carbon metabolism^20^. Further, conversion of choline, another source of one-carbon units, into glycine was downregulated (BHMT, CHDH, DMGDH, SARDH). The one-carbon metabolism was also downregulated in KIRC (Fig. 2). However, a compartment-specific change was observed in RCC subtypes. We found that genes encoding cytosolic enzymes of the folate cycle (SHMT1, MTHFR) were downregulated while mitochondrial genes (SHMT2, MTHFD2) were upregulated in KICH (Fig. 3). These mitochondrial genes were also upregulated in KIRP. On the other hand, both cytosolic and mitochondrial genes of the folate cycle were downregulated in KIRC. Further, genes involved in the methionine cycle (BHMT, MAT1A, MAT2A) and methionine salvage pathway (ADI1, AMD1, TAT) were downregulated in KICH. We also found most of these genes were downregulated in KIRP and KIRC.

### Glutathione metabolism

Serine and glycine are also precursors for glutathione synthesis. We found that extracellular glutathione metabolism was downregulated in KICH (Fig. 2). Genes of glutathione salvage pathway, gamma-glutamyltransferases (GGT1, GGT2 and GGT5), alanyl aminopeptidase (ANPEP) and glutathione S-transferases (GSTA1, GSTA5, GSTM1, GSTM2, GSTT2) were downregulated (Fig. 3). However, we observed that the gene involved in the *de novo* synthesis of glutathione was upregulated (GCLC) in KICH. This pathway requires cysteine and glutamate, which in turn might depend on the extracellular uptake of these amino acids. We found that the cysteine/glutamate transporter SLC7A11 was significantly upregulated in RCC subtypes. Further, KIRP and KIRC also showed similar alterations in glutathione metabolism. However, genes of *de novo* synthesis were unaltered in KIRC.

### Aromatic amino acid metabolism

Metabolism of aromatic amino acids was altered in RCC (Fig. 2). We observed that phenylalanine, tyrosine and tryptophan biosynthesis and tryptophan metabolism were downregulated. Phenylalanine and tryptophan are essential amino acids while tyrosine is produced *in vivo*. Phenylalanine hydroxylase (PAH) gene involved in tyrosine synthesis from phenylalanine was significantly downregulated in RCC. Genes involved in the conversion of tryptophan into serotonin and tryptamine (TPH1, DDC) were also downregulated. However, indoleamine 2, 3-dioxygenase 1 (IDO1) and tryptophan 2, 3-dioxygenase 2 (TDO2) genes involved in the first step of tryptophan/kynurenine pathway were upregulated in all three subtypes.

### Alanine, aspartate and glutamate metabolism

**Figure 4:**
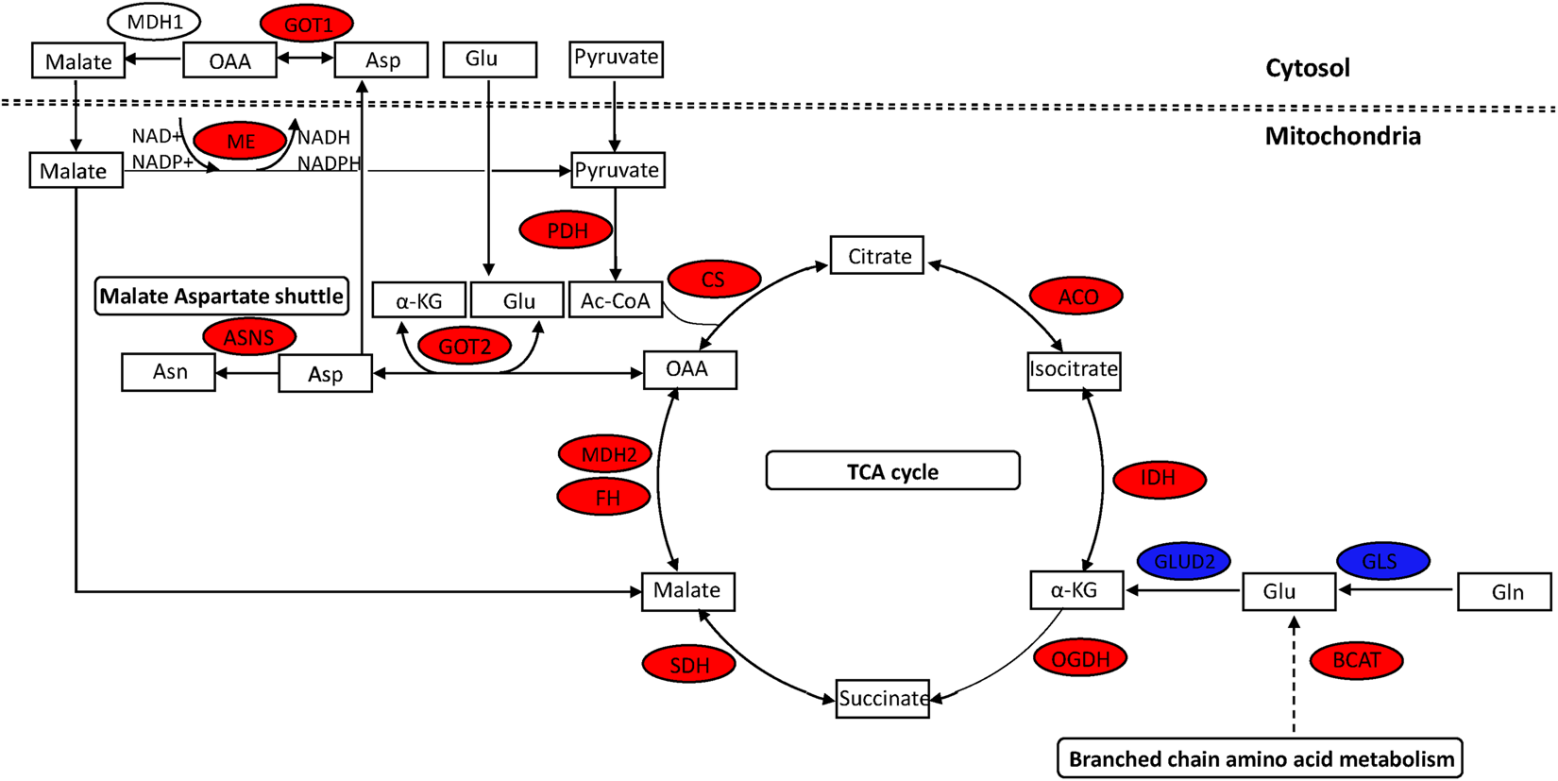
Metabolic divergence in KICH. Genes of TCA cycle, aspartate synthesis and malate-aspartate shuttle are upregulated (red) while genes involved in glutamine metabolism are downregulated (blue).

Genes involved in the conversion of alanine to pyruvate (AGXT, AGXT2, and GPT), aspartate to L-arginino-succinate (ASS1), glutamine to glutamate (GLS), glutamate to α-ketoglutarate (GLUD2) and glutamate to succinate route (GAD, GABAT, SSADH) were downregulated in RCC. Interestingly, genes involved in the interconversion of oxaloacetate and aspartate (cytosol: GOT1, mitochondrial: GOT2) were upregulated only in KICH, while downregulated in KIRC (Fig. 4). In this pathway, glutamate is converted to α-ketoglutarate and aspartate in mitochondria by GOT2 and aspartate is converted into oxaloacetate (OAA) in the cytosol by GOT1^21^. ASNS involved in the conversion of aspartate to asparagine was upregulated in RCC. Further, the gene encoding malate dehydrogenase enzyme, MDH2 was also upregulated which suggests that aspartate-malate shuttle is affected in KICH. Additionally, mitochondrial NADP-dependent malic enzyme ME3 involved in the conversion of malate to pyruvate was upregulated. This reaction is associated with NADPH production and maintenance of redox^22^. Despite overall downregulation of branched chain amino acids metabolism in RCC (Fig. 2), the expression of branched chain aminotransferase (BCAT1), the first gene of this pathway, was upregulated in RCC (Fig. 4). This reaction generates glutamate as a byproduct, which can support *de novo* glutathione biosynthesis or anaplerotic reactions.

### Arginine and proline metabolism

Genes involved in arginine and proline metabolism and polyamine synthesis were downregulated in RCC. Ornithine decarboxylase (ODC1), the rate limiting enzyme of polyamine synthesis was downregulated in KICH and KIRC. An alternative route to polyamines generation from arginine via agmatine was also affected since genes encoding arginine decarboxylase (AZIN2) and agmatinase (AGMAT) were downregulated. Further, genes that participate in the urea cycle namely nitric oxide synthase (NOS), arginase 2 (ARG2) and ornithine transcarbamylase (OTC) were also downregulated in RCC. These genes control the conversion of arginine to citrulline, arginine to ornithine and ornithine to citrulline, respectively. However, in KICH, we observed that OTC was upregulated.

### Central carbon metabolism

Glycolysis/ gluconeogenesis pathway and fructose and mannose metabolism were upregulated only in KIRP and KIRC. HIF1A target genes of the glycolytic pathway (GLUT1, HK2, HK3, ALDOA, GAPDH, PGK1, ENO1, LDHA, and PDK1) were upregulated. On the other hand, the TCA cycle and oxidative phosphorylation were upregulated in KICH (Figs. 2 and 4). Further, genes involved in pyruvate to acetyl-CoA conversion (DLAT, PDH) and acetate to acetyl-CoA (ACSS1, ACSS3) conversion were upregulated in KICH and were downregulated in KIRC and KIRP. However, genes involved in the conversion of pyruvate to oxoacetate (PC) and oxoacetate to PEP (PCK1, PCK2) were downregulated in RCC. UDP glucuronosyltransferase family genes were mostly downregulated in KICH and KIRP while upregulated in KIRC. These genes participate in the interconversion of D-glucuronate and UDP-D-glucuronate. The pentose phosphate pathway, purine and pyrimidine metabolism were also upregulated in KIRP **(**Fig. 2**)**.

### Fatty acid metabolism

Fatty acid synthase (FASN) was upregulated in KICH and KIRP. Genes of fatty acid degradation, ketogenesis (HMGCS2), cholesterol metabolism (CYP7A1, CYP8B1, CYP27B1), steroid hormone synthesis, lipid transport (APOA1, APOA2 and APOA5) and carnitine shuttle were downregulated suggesting altered lipid metabolism in RCC. Further, the metabolism of xenobiotics by cytochrome P450 was also downregulated in KICH. Although most genes of this pathway were downregulated, few members of the cytochrome P450 superfamily with known links to cancer were upregulated in KICH (CYP1A1, CYP3A4, CYP3A7)^23^.

### Co-expression of metabolic genes in RCC

**Figure 5:**
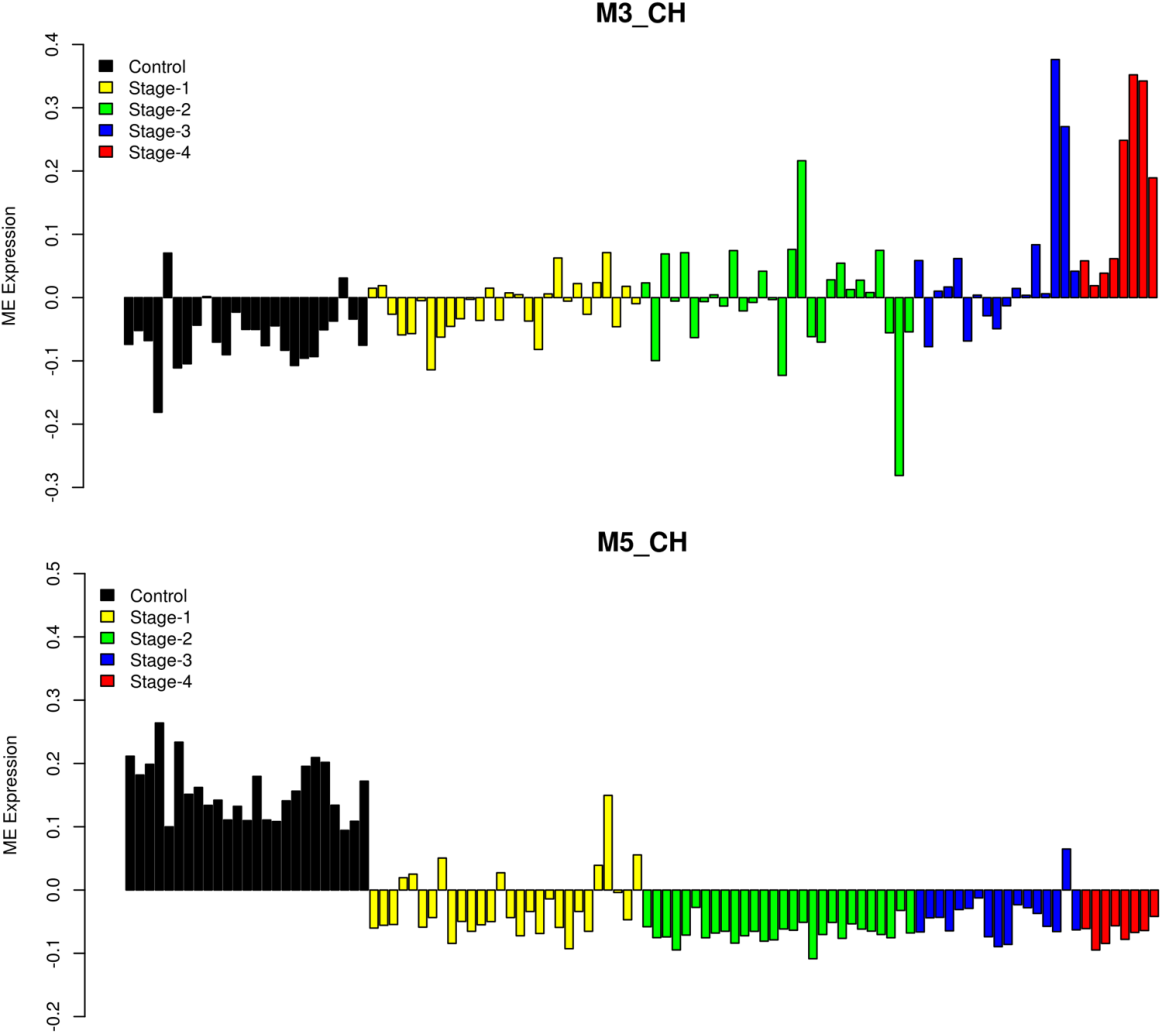
Eigengene (ME) expression profile of metabolic modules in KICH. y-axis represents expression values and x-axis represents KICH samples coloured according to the stages (normal - black, stage 1 - yellow, stage 2 - green, stage 3 - blue and stage 4 - red).

In the previous analysis, we considered only the matched normal and tumor samples to identify reporter metabolic pathways. We extended this study to include all the available samples of RCC to understand the variations within tumor samples at the level of metabolism. We performed unsupervised Weighted Gene Co-expression Network Analysis (WGCNA) to identify modules of co-expressed genes and explored their variation in a cancer-stage specific manner. We identified 7 metabolic modules in KICH which showed disease- and stage-specific changes. M5_CH, M6_CH and M7_CH modules showed a negative correlation with the disease while M1_CH, M2_CH and M3_CH modules showed a positive correlation with the disease (Table 1). The M5_CH module was downregulated in most tumor samples (Fig. 5) while M6_CH and M7_CH modules showed differences with respect to few late stage samples that resembled normal samples. Interestingly, these late stage samples mostly correspond to metabolically divergent KICH (KICH-MDD) samples reported recently (Fig. S1). Major pathways associated with each module are provided in Table 1 (see Data S2). The M5_CH module included downregulated reporter metabolic pathways. The M6_CH module was associated with protein modification and glycosphingolipid metabolism while the M7_CH module was associated with sphingolipid metabolism, and starch and sucrose metabolism. Both these modules showed a significant correlation with the overall survival time (Table 1).

**Table 1:** Correlation between module eigengene (ME) expression value and disease, stages and overall survival for KICH.

Module (Size)	Disease	Stage	Overall Survival	Pathways
M1_CH (755)	0.59 (3e-11)	0.27 (0.006)	0.31 (0.005)	Propanoate metabolism (1.9E-3), Valine, leucine, and isoleucine metabolism (3.1E-3), Tricarboxylic acid cycle and glyoxylate dicarboxylate metabolism (1.8E-2)
M2_CH (455)	0.54 (2e-9)	0.37 (9e-5)	0.34 (0.002)	Oxidative phosphorylation (1.1E-14), Nucleotide metabolism (2.2E-3), N-glycan metabolism (9.3E-3)
M3_CH (269)	0.35 (3e-4)	0.54 (2e-9)	−0.22 (0.05)	Isolated (2.8E-3), Lysine metabolism (3.5E-3), Aminoacyl-tRNA biosynthesis (8.4E-3), Chondroitin heparan sulfate biosynthesis (1.5E-2)
M4_CH (352)	−0.33 (7e-4)	−0.21 (0.03)	−0.26 (0.02)	Isolated (1.4E-12), Transport, Golgi apparatus (4.6E-4)
M5_CH (1138)	−0.9 (9e-40)	−0.72 (7e-18)	−0.18 (0.1)	Metabolism of xenobiotics by cytochrome P450 (4.6E-13), Glycine, serine and threonine metabolism (3.7E-7), Alanine, aspartate and glutamate metabolism (1.9E-4)
M6_CH (227)	−0.32 (8e-4)	−0.11 (0.2)	−0.32 (0.004)	Protein modification (2.3E-6), Chondroitin heparan sulfate biosynthesis (2.0E-3), Glycosphingolipid metabolism (4.3E-2)
M7_CH (413)	−0.69 (2e-16)	−0.39 (3e-5)	−0.44 (4e-5)	Nucleotide metabolism (2.8E-4), Sphingolipid metabolism (8.5E-3), Starch and sucrose metabolism (2.9E-2)

Pearson correlation is given with the p-value inside the bracket. HMR2 metabolic pathways associated with each module are given with corresponding p-value inside the bracket. The entire list of pathways is provided in Data S2.

**Figure 6:**
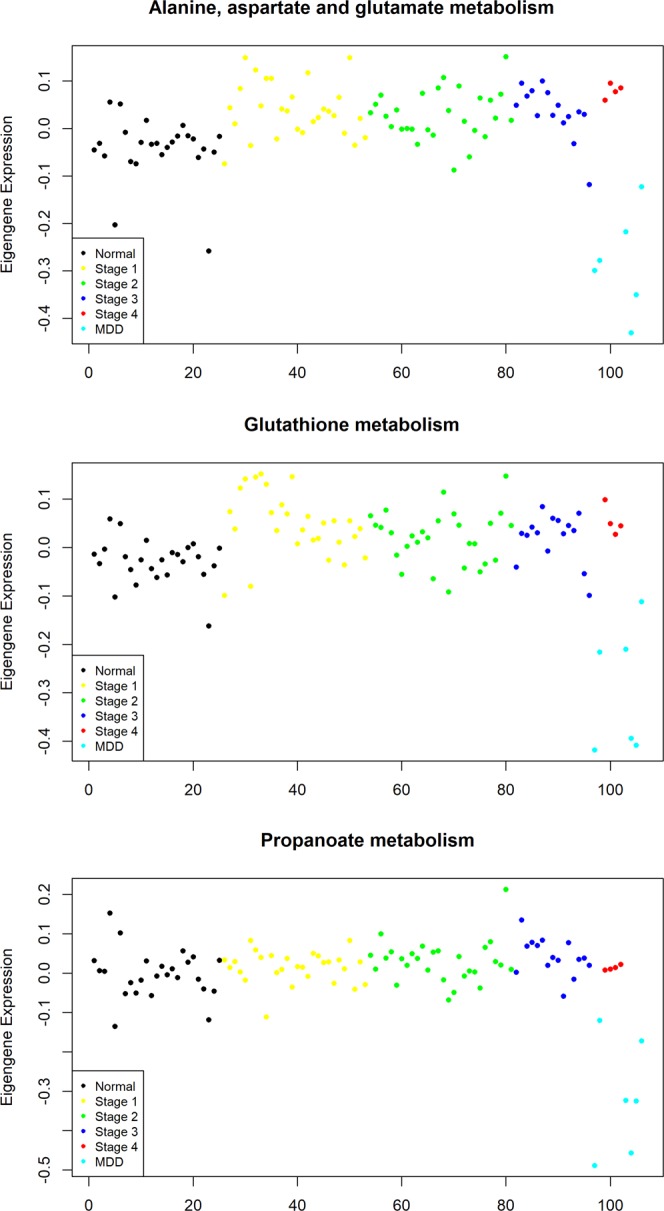
Metabolic pathways associated with KICH-MDD. Eigengene (ME) expression profile of metabolic pathways is shown. y-axis represents expression values and x-axis represents KICH samples coloured according to the stages (normal - black, stage 1 - yellow, stage 2 - green, stage 3 - blue and stage 4 - red) and subtype (MDD - cyan).

The upregulated M1_CH and M2_CH modules also showed differences with respect to KICH-MDD samples (Fig. S1). These late stage samples resembled normal samples. The M2_CH module was associated with oxidative phosphorylation while the M1_CH module was associated with propanoate metabolism, valine, leucine, and isoleucine metabolism, tricarboxylic acid cycle and glyoxylate dicarboxylate metabolism (Table 1 and Data S3). Further, this module included genes (GOT1, GOT2, BCAT1 and GCLC) that were found to be dysregulated in our study. We found that genes of glutathione metabolism, propanoate metabolism and alanine, aspartate and glutamate metabolism can distinguish KICH-MDD samples (Fig. 6). Both M1_CH and M2_CH modules showed a significant correlation with stages and overall survival time. The M3_CH module showed a higher stage-specific correlation and was associated with aminoacyl-tRNA biosynthesis and isolated reactions in HMR2 corresponding to cell cycle genes (Table 1). This module also included metabolic genes involved in pyrimidine metabolism (POLA2, RRM2, POLD1, POLE2, POLR3D, CAD, POLR3G, POLE), glycosaminoglycan metabolism (CHPF, CHPF2, B3GAT2, B3GALT6, CHSY3, CHST14), amino acid metabolism (DNMT1, SHMT2, MTHFD2, DNMT3B, TYMS, SRM, TDO2, ASNS) and lipid metabolism (FASN, ELOVL5, NRF1, FADS2, SQLE, CYP2R1, P4HB). We observed that the M3_CH module was specific to KICH-MDD.

We identified metabolic modules of KIRP that showed disease- and stage-specific changes. M1_RP, M2_RP, M3_RP and M4_RP modules showed a negative correlation with the disease while M5_RP, M6_RP, M7_RP and M8_RP modules showed a positive correlation with the disease (Table 2). KIRP samples showed a heterogeneous behavior in different modules (Fig. S2). The modules were either upregulated or downregulated in only some KIRP samples from different stages. The M2_RP module was associated with many reporter metabolic pathways and the M1_RP module was associated with the tricarboxylic acid cycle and glyoxylate dicarboxylate metabolism and oxidative phosphorylation (Table 2 and Data S2). The upregulated M5_RP module was also associated with oxidative phosphorylation suggesting a complex pattern of gene expression in this pathway. On the other hand, the M3_RP module was downregulated in most KIRP samples and is associated with O-glycan metabolism and prostaglandin biosynthesis (Fig. S2). Further, the M8_RP module was upregulated in mostly late stages of KIRP and was associated with nucleotide metabolism (RRM2, CAD, TYMS, POLA2, NT5E, NME7, POLE2, POLR2D, POLE3, POLR3G, TK1, POLE). This module also included genes linked to HIF1A transcriptional activity (LDHA, NT5E, CA9, HK2), carbohydrate metabolism (RPIA, PFKFB4, NUP107, NUP62, HAS3, NUP43, ENO2, PFKP, NUP37), one carbon metabolism (MTHFD1L, MTHFD2, DNMT3B, TYMS) and cell cycle.

**Table 2:** Correlation between module eigengene (ME) expression value and disease, stages and overall survival for KIRP.

Module (Size)	Disease	Stage	Overall Survival	Pathways
M1_RP (335)	−0.38 (9e-13)	−0.24 (4e-5)	0.0088 (0.9)	Valine, leucine and isoleucine degradation (8.3E-12), Tricarboxylic acid cycle and glyoxylate dicarboxylate metabolism (1.1E-10), Propanoate metabolism (1.9E-4), Oxidative phosphorylation (0.02)
M2_RP (428)	−0.36 (4e-11)	−0.26 (6e-6)	0.068 (0.2)	Glycine, serine and threonine metabolism (6.6E-10), Pyruvate metabolism (4.8E-6), Arginine and proline metabolism (8.3E-5), Alanine, aspartate and glutamate metabolism (8.2E-4)
M3_RP (474)	−0.82 (2e-79)	−0.3 (2e-7)	−0.084 (0.2)	O-glycan metabolism (1.6E-4), prostaglandin biosynthesis (2.3E-3), Keratan sulfate biosynthesis (5.4E-3), Estrogen metabolism (7.9E-3)
M4_RP (954)	−0.27 (6e-7)	−0.07 (0.2)	−0.033 (0.6)	Isolated (2.43E-19), Inositol phosphate metabolism (0.01)
M5_RP (618)	0.28 (2e-7)	0.17 (0.004)	−0.0048 (0.9)	Oxidative phosphorylation (2.8E-5), Nucleotide metabolism (1.4E-4), Aminoacyl-tRNA biosynthesis (1.3E-3), N-glycan metabolism (0.01), Pyrimidine metabolism (0.01)
M6_RP (417)	0.34 (5e-10)	0.011 (0.9)	−0.0051 (0.9)	Glucocorticoid biosynthesis (0.03)
M7_RP (203)	0.27 (1e-6)	0.086 (0.1)	−0.032 (0.6)	Amino sugar and nucleotide sugar metabolism (2.7E-3), Purine metabolism (0.03)
M8_RP (166)	0.5 (1e-21)	0.64 (3e-35)	−0.16 (0.005)	Nucleotide metabolism (0.01), Pyrimidine metabolism (0.02)

Pearson correlation is given with *p*-values inside the bracket. HMR2 metabolic pathways associated with each module are given with corresponding p-value inside the bracket. The entire list of pathways is provided in Data S2.

In KIRC, modules M2_RC, M3_RC and M4_RC showed a positive correlation with the disease while M1_RC, M7_RC, M8_RC and M9_RC modules showed a negative correlation with the disease (Table 3). The M4_RC module was upregulated in most late stage KIRC samples (Fig. S3). However, M2_RC and M3_RC modules were upregulated only in some KIRC samples. Major pathways associated with each module are provided in Table 3 (see Data S2**)**. The M4_RC module was associated with glycolysis and fructose and mannose metabolism. This module also included genes of cell cycle, purine metabolism and HIF1 transcriptional activity (PRKCG, PRKCB, SLC2A1, PIK3CD, ENO1, ENO2, HK2, HK1, HK3, LDHA, PGK1, ALDOA, GAPDH, PDK1). The M6_RC module showed weak correlation with stages of KIRC and it included genes of pentose and glucuronate interconversions (UDP Glucuronosyltransferase family genes) and glycine, serine and threonine metabolism (DMGDH, SHMT1, BHMT, BHMT2, CHDH, SARDH). Further, M8_RC and M9_RC modules were downregulated in most KIRC samples. The M8_RC module was associated with protein modification and glycine, serine and threonine metabolism while the M9_RC module was associated with tricarboxylic acid cycle and glyoxylate dicarboxylate metabolism and other reporter metabolic pathways.

**Table 3:** Correlation between module eigengene (ME) expression value and disease, stages and overall survival for KIRC.

Module (Size)	Disease	Stage	Overall Survival	Pathways
M1_RC (315)	−0.3 (1e-13)	−0.058 (0.2)	0.0044 (0.9)	Oxidative phosphorylation (1.1E-15), Arachidonic acid metabolism (2.1E-3), prostaglandin biosynthesis (2.3E-3), Pentose and glucuronate interconversions (3.1E-3)
M2_RC (516)	0.29 (4e-13)	0.32 (2e-15)	−0.086 (0.05)	Nucleotide metabolism (9.5E-3), Aminoacyl-tRNA biosynthesis (1.8E-2)
M3_RC (256)	0.31 (7e-15)	0.24 (5e-9)	−0.2 (4e-6)	Glucocorticoid biosynthesis (6.1E-4), Starch and sucrose metabolism (1.9E-2), Lysine metabolism (1.5E-2)
M4_RC (422)	0.77 (3e-118)	0.56 (8e-49)	−0.11 (0.01)	Fructose and mannose metabolism (2.2E-2), Glycolysis/Gluconeogenesis (2.3E-2), Porphyrin metabolism (3.8E-2)
M5_RC (228)	0.18 (8e-6)	−0.043 (0.3)	0.033 (0.5)	Protein modification (1.8E-8), Chondroitin heparan sulfate biosynthesis (6.0E-6), Purine metabolism (2.0E-3)
M6_RC (323)	−0.063 (0.1)	−0.13 (0.001)	0.14 (0.001)	Metabolism of xenobiotics by cytochrome P450 (1.3E-7), Glycine, serine and threonine metabolism (2.6E-5), Pentose and glucuronate interconversions (3.7E-4).
M7_RC (728)	−0.28 (7e-12)	−0.28 (8e-12)	0.034 (0.4)	Isolated (4.9E-25), Transport, Golgi apparatus (1.3E-3), Inositol phosphate metabolism (0.01)
M8_RC (465)	−0.9 (1e-210)	−0.48 (9e-36)	−0.076 (0.09)	Protein modification (9.8E-3), Serotonin and melatonin biosynthesis (9.8E-3), Glycine, serine and threonine metabolism (1.2E-2), Retinol metabolism (2.8E-2)
M9_RC (322)	−0.71 (5e-91)	−0.48 (2e-34)	0.12 (0.007)	Valine, leucine and isoleucine degradation (1.4E-16), Tricarboxylic acid cycle and glyoxylate dicarboxylate metabolism (8.9E-12), Alanine, aspartate and glutamate metabolism (6.2E-7)

Pearson correlation is given with *p*-values inside the bracket. HMR2 metabolic pathways associated with each module are given with corresponding p-value inside the bracket. The entire list of pathways is provided in Data S2.

## Discussion

Identifying the shared and unique features of RCC subtypes is important for differentiating subtypes and for an effective treatment. Different evidences suggest that cancer cells reprogram the metabolism to meet the requirement of cell growth and division. This presents opportunities for cancer diagnosis and treatment based on metabolic biomarkers and targets, respectively. In this work, we have performed the metabolic network analysis of RCC subtypes to reveal the systems-level alterations. In addition to metabolic changes, we also studied the co-expression pattern of metabolic genes in each sample to understand the variations in RCC metabolism.

We found that amino acids: glycine, serine and threonine metabolism (one-carbon metabolism), alanine, aspartate and glutamate metabolism, aromatic amino acid and branched chain amino acid metabolism were downregulated in RCC compared to tumor-matched normal samples (Fig. 2**)**. One carbon metabolism fuels the synthesis of amino acids, nucleotides, and polyamines, regulates the gene expression epigenetically and maintains redox homeostasis through methionine cycle^24,25^. We also found that the polyamine synthesis pathway was downregulated in RCC. However, studies have shown that the gene expression and metabolites of one-carbon metabolism are upregulated only in aggressive KIRC^11,12^. Polyamines regulate cell proliferation and its levels are high in multiple cancers^26,27^. These differences can be attributed to tumor or stage-specific differences. We found that the expression of genes in glutathione (GSH) metabolism was dysregulated in RCC, which can affect the GSH levels and sensitivity to the oxidative stress. Our observations are consistent with recent studies focusing on glutathione metabolism in KICH^28,29^. We also observed that the pentose phosphatase pathway genes were upregulated in RCC. The pentose phosphatase pathway intermediates are shown to be high in a metabolomic study of KIRC^11^. Although aromatic amino acid metabolism was downregulated in RCC, the tryptophan/kynurenine pathway genes (TDO1 and IDO1) were upregulated. Kynurenines have an immunoregulatory role of restricting the T cell activation^30^. UDP glucuronosyltransferase family of genes were differentially expressed in RCC subtypes. These genes are shown to be dysregulated to a different extent and in different directions across cancers^4^.

The canonical route to generate glutamate from glutamine for anaplerotic reactions was also downregulated **(**Fig. 4**)**. However, the upregulation of glutamic-oxaloacetic transaminase enzymes GOT1 and GOT2 in KICH suggest a non-canonical route to utilize the carbon and nitrogen derived from glutamine (Fig. 4**)**. Coloff *et al*.^31^ have shown that the upregulation of transaminases and downregulation of GLUD can promote glutamine anaplerosis and non-essential amino acids (NEAA) synthesis in proliferating mammary epithelial cells. Further, GOT1 and GOT2 can trigger a series of reactions involving the conversion of aspartate to pyruvate. This can potentially play a role in maintaining the redox state by increasing NADPH/NADP^+^ ratio. Human pancreatic ductal adenocarcinoma (PDAC) relies on the pathway involving GOT1 and knockdown of it is shown to increase reactive oxygen species and a decrease in growth^32^.

An increase in the expression of GOT1/2 and mitochondrial genes in only KICH suggests metabolic divergence. KIRC and KIRP showed an increase in the expression of genes in glycolytic pathway and fructose and mannose metabolism. The upregulated metabolic genes in KIRC and KIRP were linked to HIF1A, while in KICH were linked to the cell cycle transcriptional activators E2F1 and FOXM1 (Table S4). von Hippel-Lindau tumor suppressor (VHL) loss and HIF1A stabilization is the hallmark of KIRC^12^. Further, HIF1A is shown to inhibit the flux from glycolysis to the TCA cycle and promote glutamine reductive carboxylation (reverse TCA flux) for citrate generation. Interestingly, HIF1A is also shown to suppress the expression of aspartate producing genes GOT1 and GOT2^33^. We also found argininosuccinate synthase 1 (ASS1) expression was downregulated, which can increase aspartate availability and is associated with poor prognosis in multiple cancers^34,35^. In RCC, an increase in aspartate levels can promote cell proliferation due to its role in nucleotide synthesis^36^. In KICH, genes related to the aspartate-malate shuttle were also upregulated suggesting that aspartate can act as an anaplerotic source for the TCA cycle. Further, FOXM1 and its targets (ASNS and FASN) were upregulated in RCC^37^. ASNS promotes the synthesis of asparagine, which is shown to be a suppressor of apoptosis in response to the glutamine withdrawal^38^. FASN has an important role in tumor growth and survival^39^. On the other hand, the down regulated metabolic genes are associated with HNF4A, PPAR and LXR (Table S3). HNF4A is a proximal tubule specific transcription factor and is downregulated in late stages of KIRP and KIRC^7^. PPAR and LXR are nuclear receptors involved in the regulation of lipid metabolism^40,41^.

The co-expression pattern of metabolic genes showed that most metabolic changes in KICH-MDD are similar to other KICH samples and normal samples. Mitochondrial/oxidative metabolism was downregulated in MDD compared to other KICH samples consistent with the previous observation^10^. Genes of glutathione metabolism, propanoate metabolism and alanine, aspartate and glutamate metabolism were also differentially expressed in KICH-MDD. AMPK-mTOR signaling involved in mitochondrial biogenesis is shown to be dysregulated in KICH^42^. We observed that the expression of components of the AMPK complex was significantly upregulated in KICH samples compared to MDD samples (PRKAA2, PRKAB1, PRKAG1, PRKAG2). On the other hand, we found that a module related to cell cycle, pyrimidine metabolism and amino acid metabolism (M3_CH) showed positive correlation with stages of KICH and was specific for the MDD group. The mitochondrial one-carbon metabolic genes of this module were upregulated. This pathway helps to maintain the mitochondrial redox homeostasis during tumor growth^43^. The MDD group also consists of samples that were reclassified as KICH from KIRC and these samples displayed the characteristics of the HIF1A cluster with its targets upregulated (e.g. CA9). These observations suggest that MDD samples have low AMPK and mitochondrial activity and high cell cycle and HIF1A activity. These features can be related to the aggressiveness of RCC samples. A similar classification of hepatocellular carcinoma (HCC) samples into HIF1A and AMPK clusters with the more aggressive stage belonging to the HIF1A cluster has been shown^44^. The active and functional form of mitochondria has been associated with a less aggressive form of tumors. Damaged mitochondria lead to enhanced ROS production and a higher mutational load^45^. We also found a module related to cell cycle and HIF1 transcriptional activity was upregulated in late stage samples of KIRC and KIRP, which can serve as a biomarker for staging. Although KIRC and KICH show distinct metabolic phenotypes (glycolytic and oxidative), KIRP showed a more heterogeneous behavior. In KIRP, the mitochondrial metabolism was not fully downregulated. This can represent a hybrid phenotype with a subclass of samples showing aggressive phenotype like KIRC and less aggressive phenotype like chromophobe. A hybrid metabolic phenotype utilizing both glycolysis and oxidative phosphorylation is shown to exist based on the mutual antagonism between HIF1 and AMPK^46^.

In summary, our work not only confirmed the previous findings on RCC metabolism^9–12^ but also further explored the metabolic differences between RCC subtypes. We specifically showed the metabolic divergence of KICH compared to other subtypes and linked the subtype-specific metabolic changes to the difference in the transcriptional regulation. The co-expression of metabolic genes showed the pattern of gene expression in each patient. KICH showed uniform metabolic changes compared to KIRC and KIRP across stages except for the MDD samples. We also found co-expression modules that showed tumor stage-specific behavior. Thus, our study identifies metabolic features associated with RCC subtypes, which can help towards cancer diagnosis and prognosis. Presently, positron emission tomography (PET) imaging with the glucose analogue 18F-fluorodeoxyglucose (18F-FDG) and 18F-glutamine is used to detect altered glucose uptake and glutamine metabolism in RCC, respectively^36,47,48^. Isotope tracers such as ^13^C are also used in the reconstruction of metabolic pathways in cancer^49,50^. ^13^C-glucose based tracer study showed the metabolic reprogramming in Fumarate hydratase (FH)-deficient renal cancer^51^. Further, defective mitochondria in RCC can impose tumor transformation by deuterium (heavy hydrogen) oncoisotope accumulation^52^. Therefore, extracellular deuterium depletion (deupletion) can act as a metabolic therapeutic adjuvant and deupletion can be initiated via diet and potables in integrative therapeutic settings^52,53^. These studies warrant consideration of altered metabolism to treat RCC. Furthermore, metabolic alterations identified by integration of genome-scale and transcriptomic data of less common RCC subtypes have to be supported by global metabolomic profiling to explore new opportunities for diagnostic and therapeutic intervention.

## Methods

In this study, TCGA pan-cancer RNA-Sequencing (RNA-Seq) data were obtained from Genomic Data Commons (GDC) portal (https://gdc.cancer.gov/). The pan-cancer atlas includes preprocessed gene expression data of 20531 genes from 33 cancer types. We restricted our analysis to 14 cancer-types that have at least 15 tumor-adjacent normal samples for clustering the cancer types based on the metabolic differences. Table S1 shows the number of matched normal and tumor samples for each cancer type. The TCGA sample barcode scheme was used to map the tumor and tumor-adjacent normal samples. The human genome-scale metabolic model (HMR version 2.0) was used to study the cancer metabolism. HMR2 is a comprehensive model with 8181 reactions, 3161 unique metabolites, and 3765 genes. The log fold-change difference in HMR2 gene expression between matched normal and tumor samples was calculated and top 10% genes with high variance across samples (361 genes) were used for the PCA.

To specifically map the metabolic changes of RCC subtypes, the RNA-Seq raw count data of KIRC, KIRP and KICH were obtained from the GDC portal. We used only pairs of tumor and tumor-adjacent normal samples (25 for KICH, 32 for KIRP and 72 for KIRC) to perform differential gene expression analysis of HMR2 genes for each subtype using DESeq2, which also performs normalization internally using the median of ratios method^54^. Benjamini-Hochberg method was used to adjust the p-value of genes obtained in the DESeq2 analysis. The adjusted p-value of genes was used to integrate the gene expression and the genome-scale metabolic model to identify reporter metabolites by the reporter metabolite algorithm (RMA)^18^. This method transforms the p-values into Z-scores using the inverse normal distribution function and scores a metabolite based on aggregating Z scores of its k neighboring genes:

1 $${Z}_{metabolite}=\frac{1}{\sqrt{k}}\mathop{\sum }\limits_{i=1}^{k}{Z}_{i}$$

The Z score of each metabolite was corrected for background distribution (Eq. 2). 100000 sets of k genes were chosen at random to compute mean (µ_k_) and standard deviation (σ_k_).

2 $${Z}_{metabolite}^{corrected}=\frac{({Z}_{metabolite}-{\mu }_{k})}{{\sigma }_{k}}$$

Corrected Z scores were used for the p-value calculation. This method assumes that genes linked to the metabolite are co-expressed. Further, reporter pathways were also identified by aggregating the score of n metabolites (Eq. 3) of a pathway^19^.

3 $${Z}_{pathway\,}^{m}=\frac{1}{\sqrt{n}}\mathop{\sum }\limits_{metabolite=1}^{n}{Z}_{metabolite}$$

We identified the direction of change of reporter pathways by performing the analysis with only upregulated or downregulated genes in each subtype. Since RMA was performed between only tumor and tumor-adjacent normal samples, we extended our study to analyze the co-expression pattern of metabolic genes in all available RCC samples (81 KICH, 290 KIRP and 518 KIRC samples) in the GDC portal. This was done to understand the variations within the tumor samples and to map the tumor-stage specific changes. Co-expression networks of HMR2 genes were constructed for KICH, KIRC, and KIRP by performing the WGCNA in R^55^. WGCNA organizes the co-expressing genes into modules of functional pathways. Pearson correlations between gene expression levels were computed to construct the correlation matrix. The sign of correlations was retained by performing a linear transformation:

4 $${S}_{ij}=\frac{1+cor|{x}_{i},{x}_{j}|}{2}$$

A weighted adjacency matrix was constructed using a function ${a}_{ij}={S}_{ij}^{\beta }$, where β represents soft-threshold power that is calculated by a scale-free topology criterion. We obtained β = 14 for KIRC and β = 12 for KIRP and KICH. A topological overlap matrix (TOM) was obtained from the adjacency matrix and hierarchical clustering was performed using a distance measure 1-TOM^56^. Modules of minimum size 100 were identified using dynamic tree cut algorithm^57^. The module eigen-gene (ME) expression value was obtained using Singular Value Decomposition (SVD). Pearson’s correlation between ME value and clinical traits: disease (normal-0, tumor-1), stage (normal-0, stage I-1, stage II-2, stage III-3, stage IV-4) and survival data, was calculated to identify relevant tumor modules^55^. We performed hypergeometric test to identify HMR2 metabolic pathways associated with the modules. Eigengene expression values of individual pathways of significant modules were also visualized to confirm the stage-specific changes. The transcriptional factor enrichment analysis of differentially expressed genes was performed using Enrichr^17^. The upregulated and downregulated genes (adjusted p-value ≤ 0.05) between matched normal and tumor samples of each subtype were used as target genes. Enrichr provides different gene-set libraries to identify transcription factor from the target gene list. We used multiple libraries including ChEA and ENCODE_and_ChEA_Consensus_TFs to identify transcriptional factors associated with upregulated and downregulated genes.

## Supplementary information

Supplementary Information.

Supplementary Information2.

Supplementary Information3.
