# Supplementary material for: Network-based metabolic characterization of renal cell carcinoma
Source: Sci Rep. 2020 Apr 6;10:5955. doi: 10.1038/s41598-020-62853-8 (PMC7136214; doi:10.1038/s41598-020-62853-8)
Supplement: Supplementary file 1 — Supplementary Information. [file 41598_2020_62853_MOESM1_ESM.docx]

**Supplementary information**

**Network-based metabolic characterization of renal cell carcinoma**

Nishtha Pandey^1^, Vinay Lanke^1,2^ and P K Vinod^1^*

^1^Centre for Computational Natural Sciences and Bioinformatics, International Institute of Information Technology, Hyderabad-500032, India

^2^TCS Innovation Labs, Hyderabad, India

*Correspondence: [vinod.pk@iiit.ac.in](mailto:vinod.pk@iiit.ac.in)

**Table S1:** Number of tumor and tumor-adjacent normal samples of 14 cancer types used for the principal component analysis. BLCA - Bladder Urothelial Carcinoma, BRCA - Breast invasive carcinoma, COAD - Colon adenocarcinoma, HNSC - Head and Neck squamous cell carcinoma, KICH - Kidney Chromophobe, KIRC - Kidney renal clear cell carcinoma, KIRP - Kidney renal papillary cell carcinoma, LIHC - Liver hepatocellular carcinoma, LUAD - Lung adenocarcinoma, LUSC - Lung squamous cell carcinoma, PRAD - Prostate adenocarcinoma, STAD - Stomach adenocarcinoma, THCA - Thyroid carcinoma, UCEC - Uterine Corpus Endometrial Carcinoma.

| **TCGA**  **Project** | **BLCA** | **BRCA** | **COAD** | **HNSC** | **KICH** | **KIRC** | **KIRP** | **LIHC** | **LUAD** | **LUSC** | **PRAD** | **STAD** | **THCA** | **UCEC** |
| --- | --- | --- | --- | --- | --- | --- | --- | --- | --- | --- | --- | --- | --- | --- |
| **Normal** | 19 | 113 | 40 | 43 | 25 | 72 | 32 | 50 | 58 | 51 | 52 | 32 | 59 | 22 |

**Table S2:** Differentially expressed genes (|log_2_(FC)| ≥ 1 and adjusted p-value ≤ 0.05) between normal and cancer samples.

| **RCC subtype** | **No. of up regulated genes** | **No. of down regulated genes** |
| --- | --- | --- |
| KICH | 516 | 692 |
| KIRC | 413 | 579 |
| KIRP | 378 | 483 |

**Table S3:** Transcription factor enrichment of downregulated genes using Enrichr.

| **Library** | **Terms** | **Gene coverage** | **Transcription**  **Factor (TF)** | **KICH**  **(adj**  **p-val)** | **KIRC**  **(adj**  **p-val)** | **KIRP**  **(adj**  **p-val)** |
| --- | --- | --- | --- | --- | --- | --- |
| ARCHS4_TFs_Coexp | 1724 | 25983 | HNF4A | 2.37E-34 | 2.31E-15 | 1.97E-17 |
| ChEA_2016 | 645 | 49238 | HNF4A | 1.18E-17 | 1.68E-20 | 1.91E-15 |
|  | | | LXR | 7.27E-17 | 9.34E-15 | 5.36E-14 |
|  |  |  | RXR | 2.01E-17 | 8.90E-16 | 7.10E-15 |
|  |  |  | PPARA | 1.39E-11 | 4.23E-11 | 1.27E-09 |
| ENCODE_and_ChEA_Consensus_TFs_from_ChIP-X | 104 | 15562 | HNF4A | 4.00E-10 | 4.11E-06 | 5.41E-06 |
| TF_Perturbations_  Followed_by_Expression | 1958 | 19741 | HNF4A | 2.18E-29 | 1.69E-30 | 1.20E-27 |
|  | | | PPARA | 1.18E-22 | 1.17E-34 | 2.53E-24 |

**Table S4:** Transcription factor enrichment of upregulated genes using Enrichr.

| **Library** | **Terms** | **Gene coverage** | **Transcription**  **Factor (TF)** | **KICH**  **(adj**  **p-val)** | **KIRC**  **(adj**  **p-val)** | **KIRP**  **(adj**  **p-val)** |
| --- | --- | --- | --- | --- | --- | --- |
| ARCHS4_TFs_Coexp | 1724 | 25983 | HIF1A | ------ | 0.003 | 0.0015 |
|  | | | E2F1 | 1.87E-09 | 5.77E-07 | 5.05E-16 |
|  |  |  | FOXM1 | 1.16E-11 | 5.1E-08 | 3.37E-16 |
| ChEA_2016 | 645 | 49238 | HIF1A | ------ | 8.83E-07 | ------ |
|  | | | E2F1 | 4.56E-07 | ------ | ------ |
|  |  |  | FOXM1 | 0.030571 | ------ | 0.0415 |
| ENCODE_and_ChEA_Consensus_TFs_from_ChIP-X | 104 | 15562 | E2F1 | 3.12E-10 | ------ | 3.12E-06 |
| TF_Perturbations_  Followed_by_Expression | 1958 | 19741 | HIF1A | ------ | 3.29E-10 | 2.01E-04 |
|  | | | E2F1 | 1.18E-07 | 1.34E-04 | 1.63E-06 |
|  |  |  | FOXM1 | 7.02E-06 | ------ | 0.0148 |

**
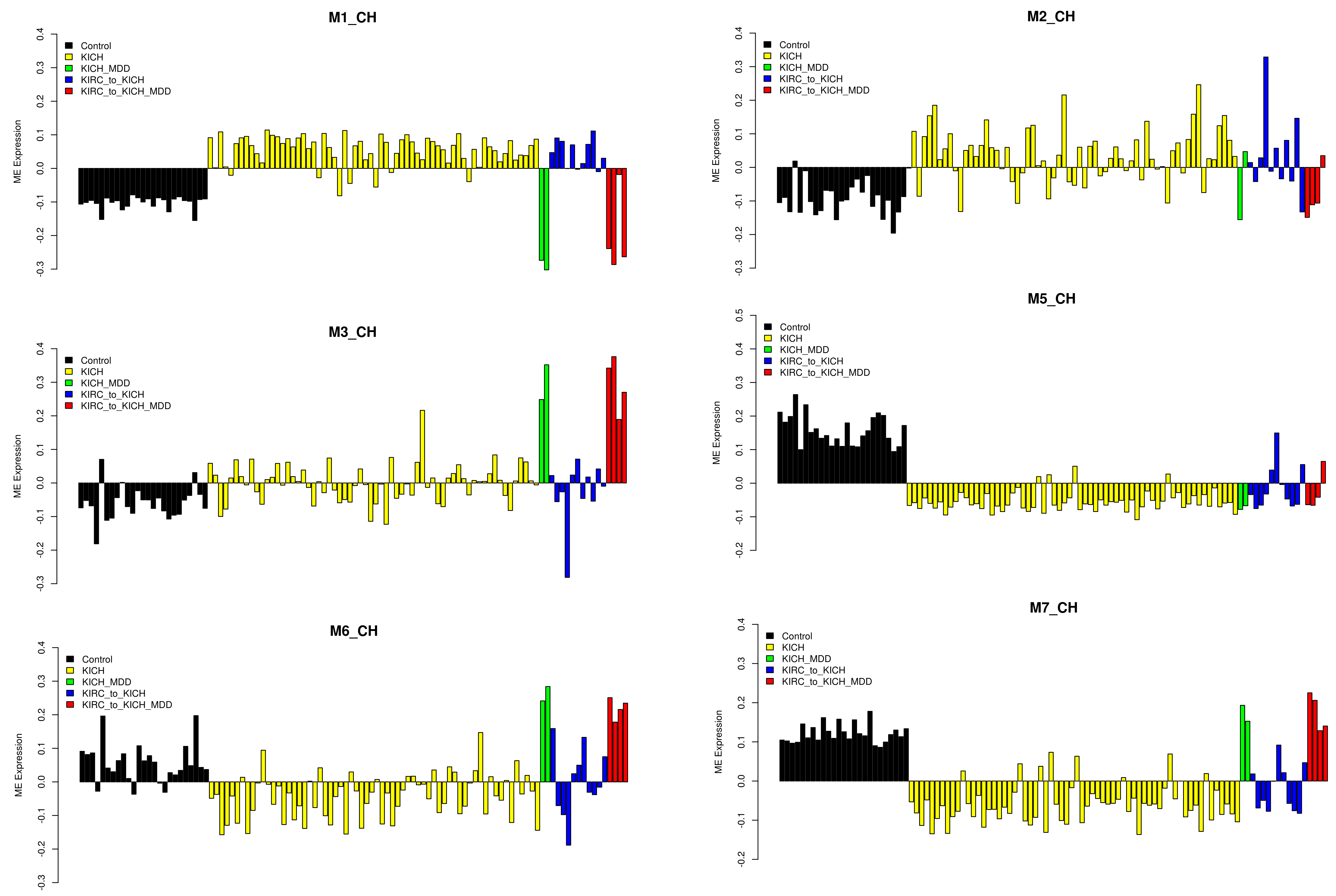
**

**Figure S1:** Eigengene (ME) expression profile of metabolic modules in KICH-MDD. y-axis represents expression values and x-axis represents KICH samples colored according to groups (KICH normal - black, tumor - yellow, MDD - green, KIRC to KICH reclassified - blue and KIRC to KICH-MDD reclassified - red).


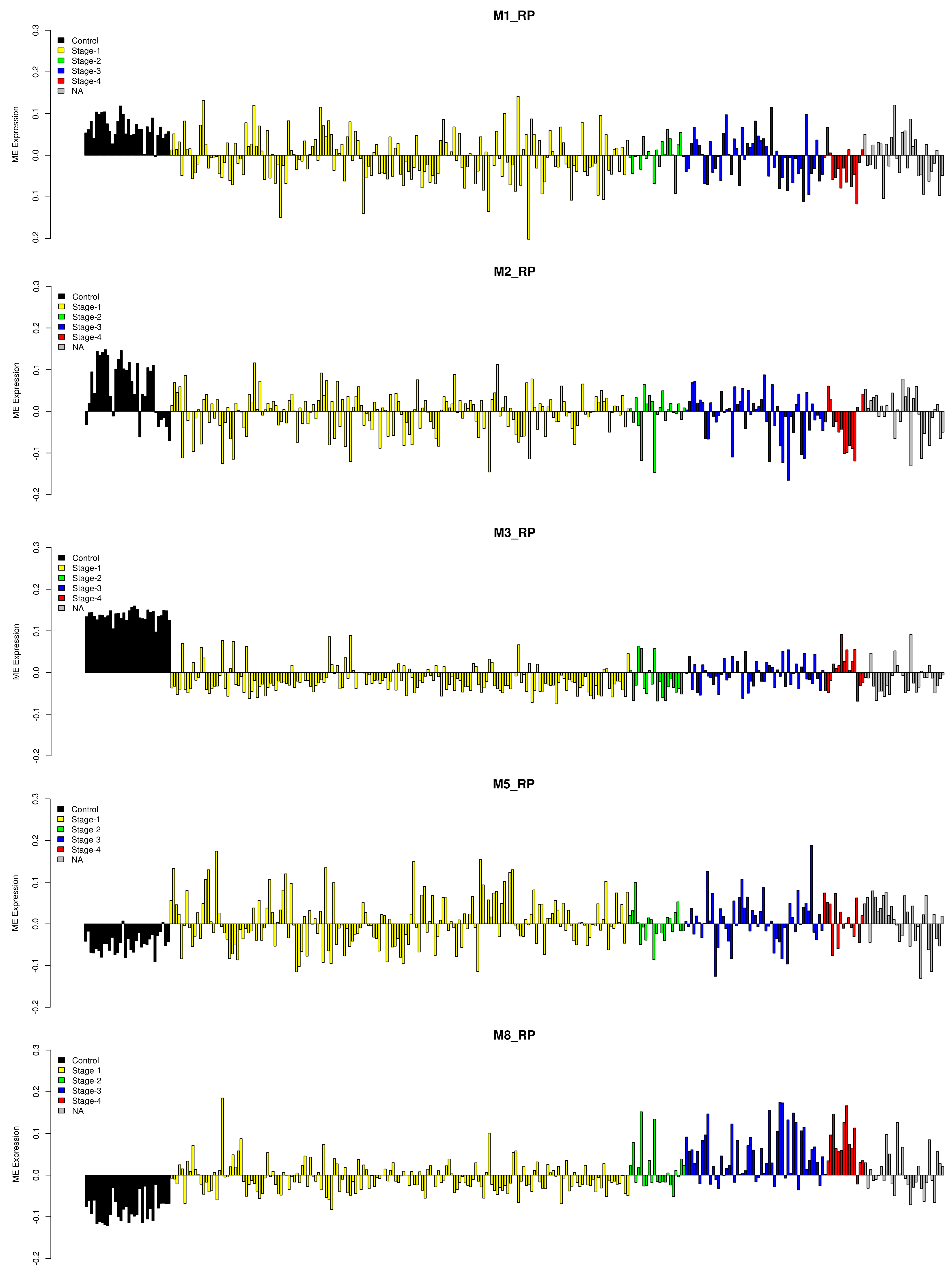


**Figure S2**: Eigengene (ME) expression profile of metabolic modules in KIRP. Though KIRP tumor samples show heterogeneous profile, the M8_RP module shows a significant late stage specific change. y-axis represents expression values and x-axis represents KIRP samples colored according to stages (KIRP normal - black, stage 1 - yellow, stage 2 - green, stage 3 – blue, stage 4 – red, not assigned - grey).

**
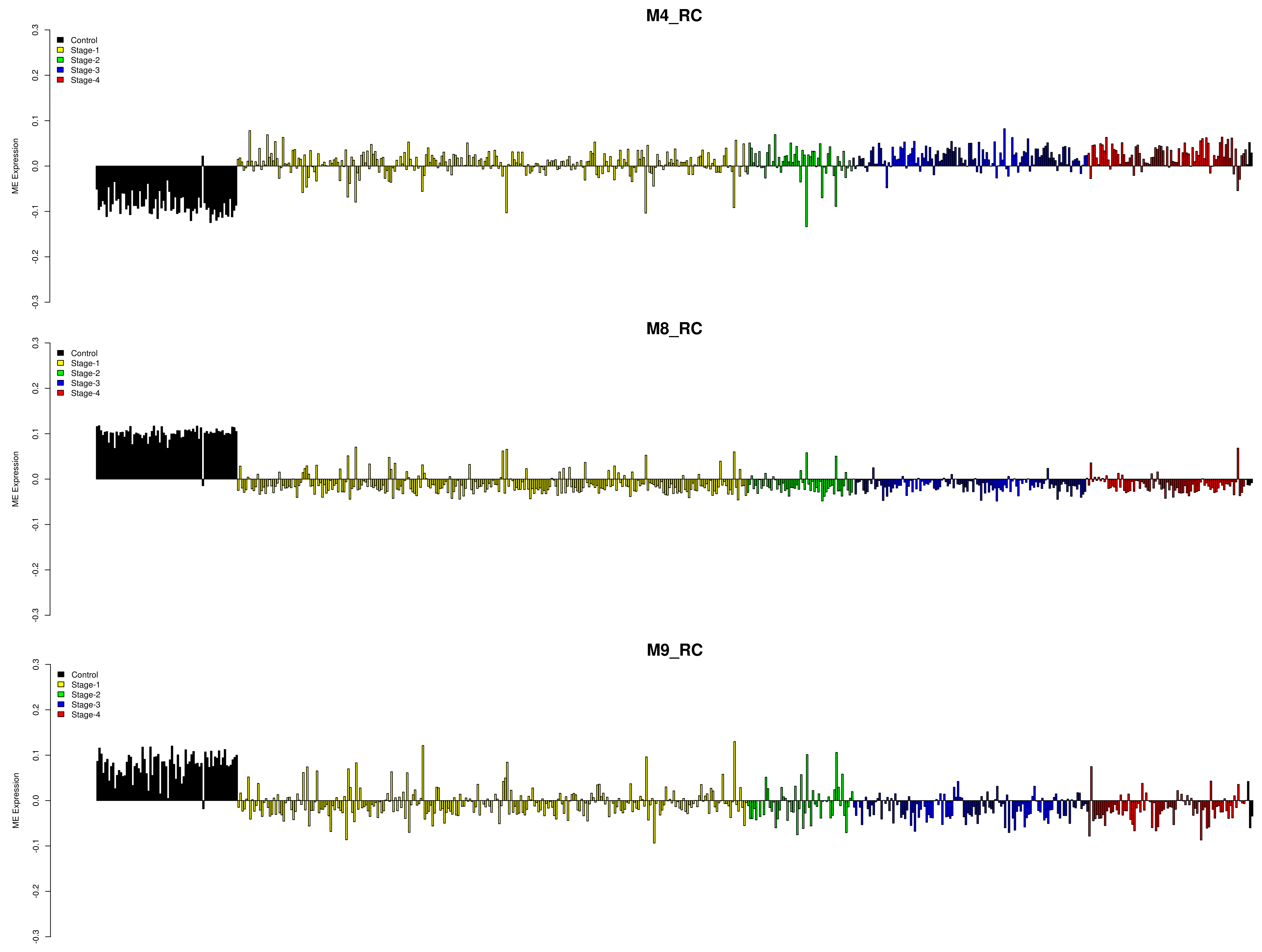
**

**Figure S3:** Eigengene (ME) expression profile of metabolic modules in KIRC. M4_RC and M9_RC modules are late stage specific modules. The M4_RC module correlates positively with stage while the M9_RC module shows negative correlation. y-axis represents expression values and x-axis represents KIRC samples colored according to stages (KIRC normal - black, stage 1 - yellow, stage 2 - green, stage 3 – blue, stage 4 – red).
